# Total body irradiation-based conditioning versus chemotherapy before allogeneic stem cell transplantation for adults with acute lymphoblastic leukemia

**DOI:** 10.46989/001c.120991

**Published:** 2024-07-12

**Authors:** Nour Ben Abdeljelil, Rihab Ouerghi, Insaf Ben Yaiche, Sabrine Mekni, Lamia Torjemane, Dorra Belloumi, Rimel Kanoun, Ines Turki, Chiraz Nasr-Ammar, Saloua Ladeb, Tarek Ben Othman

**Affiliations:** 1 Hematology and Graft National Bone Marrow Transplantation Center; 2 Hematology and Graft National Bone Marrow transplantation Center; 3 Radiotherapy Salah Azaiez Institute

**Keywords:** Acute lymphoblastic leukemia, allogeneic stem cell transplantation, myeloablative conditioning regimen, total body irradiation, relapse, survival

Total body irradiation (TBI) is used most often as part of preparative regimens in allogeneic stem cell transplantation (allo-HSCT) for acute lymphoblastic leukemia (ALL). However, high doses of TBI result in long-term toxicity and secondary malignancies. The FORUM study showed better survival and lower relapse risk with TBI-based conditioning regimen compared with chemotherapy-based (CT) regimen in pediatric patients with ALL.^1^ However, improving outcomes with TBI-based conditioning regimen in adults ALL is still a matter of debate and controversy. For logistical reasons, the use of TBI in all patients with ALL could not be applied in our or in other centers. To circumvent these constraints, we have used CT-based regimen, either busulfan (iv) and Cy or fludarabine and busulfan (iv) and Cy or thiotepa and busulfan (iv) and fludarabine in patients who could not receive TBI. We conducted a retrospective study to compare outcomes of TBI and CT based-regimens in adult ALL patients (≥18 years) undergoing allo-HSCT from HLA-matched sibling donor (MSD) between November 2003 and May 2022.

TBI-based conditioning regimen consisted of once-a-day fractionated TBI (F-TBI) with a total dose of 9.9 Gy (3.3 Gy per day) in three fractions for 3 consecutive days (d-7 until d-5 of stem cell infusion), followed by either Etoposide (60 mg/kg per day in d-3), or Cy (60 mg/kg per day for two days: d-3 and d-2). TBI was performed by a linear accelerator. The total dose delivered was 9.9 Gy to the whole body to the mid-plane of the abdomen at an instantaneous dose rate of 4.5 cGy/mn and ranged from 2.8 to 3.94 cGy/mn (mean 3.43 cGy/mn). The dose delivered to the lungs was 9 Gy with protection by a custom cache cerrobend during the second session of irradiation. Chemotherapy-based regimen consisted of either busulfan (iv) 4-times daily (0.8 mg/kg every 6 hours x16 doses) or once-daily in a 3-hour infusion (3.2 mg/kg x 4 days) and Cy 60mg/kg for two days (Bu-Cy) or fludarabine 30 mg/m^2^ once a day for 6 days and busulfan 4-times daily (0.8 mg/kg every 6 hours x 12 doses) or once-daily in a 3-hour infusion (3.2 mg/kg x 3 days) and Cy 60mg/kg for two days (FBC) or thiotepa 5 mg/kg once a day for 2 days and busulfan once-daily in a 3-hour infusion (3.2 mg/kg x 3 days) and fludarabine 50 mg/m^2^ once a day for 3 days (TBF). Graft-versus-host disease (GVHD) prophylaxis consisted on cyclosporin and a short course of methotrexate. Rabbit antithymocyte globulin has been used for a few patients with high-risk GVHD.

Informed consent was obtained from all the patients and the study approved by the local ethical committee.

Overall, 95 patients were enrolled with 61 patients conditioned with F-TBI-based regimen and 34 patients conditioned with CT. Characteristics are detailed in Table I. There were no differences between groups, except median time from diagnosis to allo-HSCT, median follow-up and stem cell source.

**Table 1.** Patient characteristics

**Patient characteristics**	**All patients** **(n=95)**	**TBI-based** **(n=61)**	**CT-based** **(n=34)**	**p**
**Median age (range), years**	30 (18-50)	28 (18-49)	33 (18-50)	0.11
**Sex ratio**	1.79	1.65	2.09	0.4
**Diagnosis**				0.2
B-ALL, n (%)	56 (59%)	33 (54%)	23 (67.6%)	
T-ALL, n (%)	39 (41%)	28 (46%)	11 (32.4%)	
**Cytogenetics and/or molecular risk**				0.1
High risk [Ph+]	35 (36.8%) [25 (26.3%)]	20 (32.8%) [13 (21.3%)]	15 (44.1%) [12 (35.3%)]	
Standard risk	49 (51.6%)	36 (59%)	13 (38.2%)	
Unknown	11 (11.6%)	5 (8.2%)	6 (17.7%)	
**Median time diagnosis-allo-HSCT (range), months**	6 (2-137)	5 (2-137)	7.5 (3-75)	**0.03**
**Disease status before transplant, n (%)**				0.4
CR1	80 (84.2%)	50 (82%)	30 (88%)	
>CR1	15 (15.8%)	11 (18%)	4 (12%)	
**MRD prior allo-HSCT (n=60)**				0.6
MRD-	27 (45%)	12 (41.4%)	15 (48.4%)	
MRD+	33 (55%)	17 (58.6%)	16 (51.6%)	
**HCT-CI score ≥2, n (%)**	15 (15.8%)	10 (16.4%)	5 (14.7%)	0.5
**EBMT score ≥2, n (%)**	59 (62%)	38 (62.3%)	21 (61.8%)	0.9
**Conditioning regimen, n (%)**				-
TBI-etoposide/cy	53 (87%) /8 (13%)	53 (87%) /8 (13%)	-	
TBF	17 (50%)	-	17 (50%)	
BU-Cy	9 (26.5%)	-	9 (26.5%)	
FBC	8 (23.5%)	-	8 (23.5%)	
**ABO mismatch, n (%)**				0.13
Matched	57 (60%)	40 (65.6%)	17 (50%)	
Major or bidirectional or minor	38 (40%)	21 (34.4%)	17 (50%)	
**Stem cell source, n (%)**				**0.05**
Bone marrow	46 (48.4%)	34 (55.7%)	12 (35%)	
Peripheral blood stem cell	49 (51.6%)	27 (44.3%)	22 (65%)	
**GVHD prophylaxis**				0.6
Cyclosporine and methotrexate+/-rATG	93 (98%)	60 (98.4%)	33 (97%)	
Cyclosporine	2 (2%)	1 (1.6%)	1 (3%)	
**Median follow-up, months (range)**	19 (2-208)	27.5 (2-208)	16 (3-96)	**0.02**

TBI:total body irradiation; CT: chemotherapy; ALL: acute lymphoblastic leukemia; Ph: Philadelphia chromosome; CR: complete remission; MRD: minimal residual disease; HCT-CI: Hematopoietic Cell Transplantation-specific Comorbidity Index; EBMT: European Society for Blood and Marrow Transplant; Cy: cyclophosphamide; TBF: thiotepa-busulfan iv-fludarabine; BU-Cy: busulfan iv-cyclophosphamide; FBC: fludarabine-busulfan iv-cyclophosphamide; rATG : rabbit antithymocyte globulin

Cumulative incidences (CI) of grade II-IV acute and extensive chronic GVHD in TBI and CT groups were 34% vs 44% and 49% vs 45% , respectively with no statistically significant difference (p=0.4 and p=0.4, respectively). The 5-year CI of Non-relapse mortality (NRM) were comparable in TBI and CT groups (27.7% and 18%, respectively, p = 0.8) (Fig 1a). The major causes of NRM were infections (n=6 vs n= 4), GVHD (n=7 vs n=1), and CMV infection (n=1 vs n=1) in TBI and CT groups, respectively. In multivariate analysis, acute GVHD identified as predictive factor of NRM (OR,11.75; 95% CI [1.28-107.29], p = 0.003). After a median follow-up of 19 months (2 months- 17 years), the 5-year CI of relapse (CIR) was significantly lower in TBI group compared to CT group (24.1% vs 48.3%, p = 0.04) (Fig 1b). In subgroup of CT-based regimen, a trend towards lower CIR was observed in TBF group (n=17) compared to other CT-based regimen (n=17) (28.4% vs 56.1%, p=0.06). In multivariate analysis, the only independent factor of CIR was CT-based regimen (OR,5.55; 95% CI [1.03-29.83], p = 0.04).

**Figure 1a.**
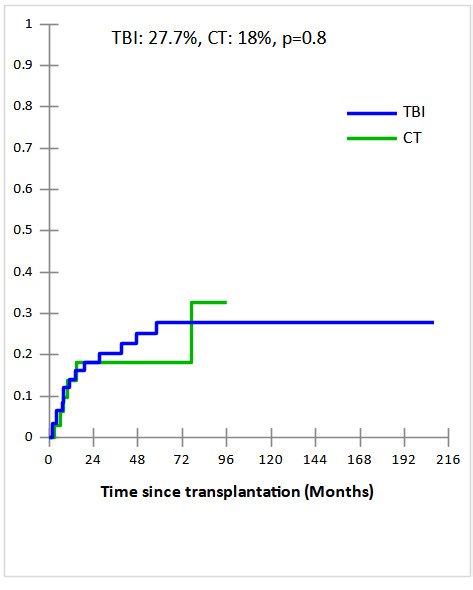
Cumulative incidence of non-relapse mortality

**Figure 1b.**
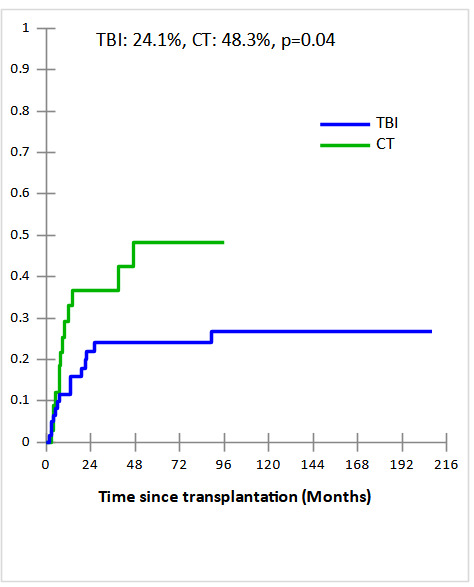
Cumulative incidence of relapse

Measurable Residual Disease (MRD) was performed by multiparameter flow cytometry (4-6 color assay) in Philadelphia chromosome-negative (Ph-) ALL and quantitative polymerase chain reaction in Ph+ ALL, BCR-ABL1 mRNA transcripts. Positive MRD was defined as MRD levels ≥ 10^-4^. MRD assessment was performed in 45.3% of patients at 3 months post allo-SCT. Among them, 58.1% achieved or maintained a negative MRD status. Nine of the 33 patients (27.3 %) who were MRD-positive at transplant converted to MRD-negative within 3 months after allo-SCT. The 5-year overall survival (OS) for TBI and CT groups were 52% and 42.6%, respectively (p=0.2) and the 5-year event-free survival (EFS) was 49% and 34.6%, respectively (p=0.1) (Fig 2a, 2b). In multivariate analysis, factors associated with worse OS were positive MRD prior allo-HSCT status (OR 3.29 95% CI [1.08-10.04], p=0.04), CT-based regimen (OR 5.23; 95% CI [1.68-16.26], p= 0.004) and acute GVHD (OR 3.26; 95% CI [1.14- 9.35], p=0.03) and with worse EFS were CT-based regimen (OR 3.37; 95% CI [1.30-8.66], p=0.01) and acute GVHD (OR 2.99; 95% CI [1.21- 7.37], p=0.02).

**Figure 2a.**
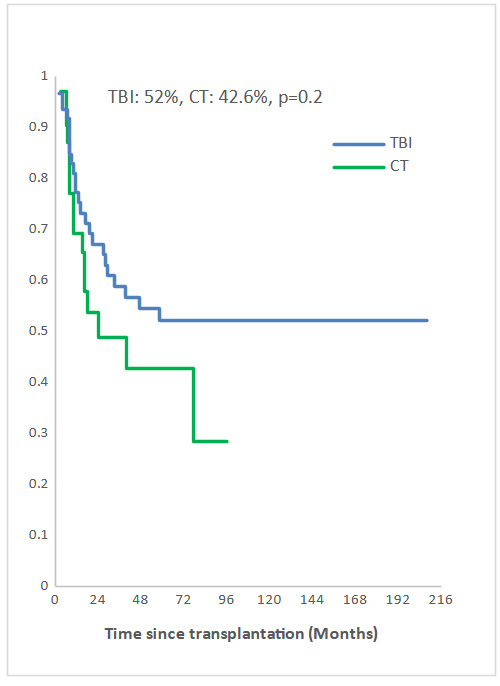
Overall survival

**Figure 2b.**
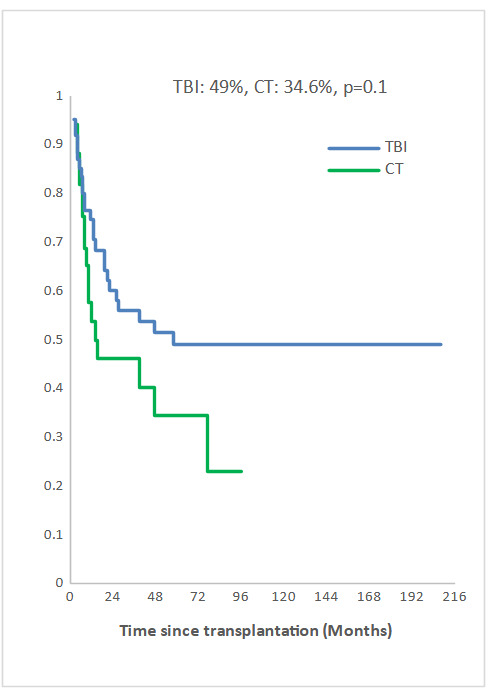
Event-free survival

Based on subgroup analysis according to MRD status before allo-HSCT, we found that MRD positive patients (n=20) who received F-TBI-based regimen had significantly better OS (p<10-4), EFS (p=0.01) and trend towards a lower CIR (10% vs 61%, p=0.06) than CT-based regimen. However, no beneficial effect of TBI in MRD negative patients (n=40) was observed.

This study is a single-centre retrospective comparison of outcomes of TBI and CT-based conditioning regimen before allo-SCT for adults with ALL. The main finding of our study was that TBI-based regimen was significantly associated with lower CIR, better OS and EFS than CT-based regimen with similar NRM.

Our findings were in line with previous studies, showing comparable NRM between TBI and CT-based regimens.^2^ The low NRM in our series could be explained by the young age of patients, the use of HLA-MSD transplants and the small number of patients transplanted with an advanced disease. In most previous studies, it has been reported that grade II-IV acute and chronic GVHD were not significantly different between TBI and CT-based.^3–6^ However, a higher incidence of grade III-IV acute GVHD was reported with TBI-based in others studies.^5,6^ TBI-based regimen was significantly associated with lower CIR than CT in uni and multivariate analysis in our cohort. This also was the conclusion of a meta-analysis of eight retrospective studies comparing TBI-based to various CT regimens in allo-HSCT for adult patients with ALL. In this systematic review, authors recommended TBI to young adult patients aged <40 years as it yields to better survival and less relapse with acceptable NRM. However, it should be noted that the scheme and total doses of TBI in these eight studies were different from ours, ranging from 8 to 14.4 Gy.^2^ The TBI conditioning in our study was mainly associated with etoposide, whereas in the major studies was TBI plus Cy. The use of TBI-etoposide was associated with lower relapse than TBI-Cy among ALL patients in CR2^7^ and in patients transplanted in CR1 or CR2.^8^

Recently a randomized study was performed in patients aged between 14 and 65 years with B-ALL standard-risk cytogenetics in first CR, demonstrated a non-inferiority for survival, relapse, and NRM in BU-Cy group compared to TBI (9Gy) and Cy, but this study was carried out in a highly selected patient and could not be extrapolated to all adult ALL.^4^

Pre-transplant positive MRD was a significant negative predictor of relapse-free survival, EFS, and OS. However, it was not associated with a higher rate of NRM.^9^ In our cohort, a positive pre-transplant MRD had a negative impact on OS without affecting the CIR, EFS or NRM. These may be explained by a small number of patients transplanted with positive MRD. Additionally, MRD was not evaluated for all patients. Conversely, based in subgroup analysis, a trend towards lower CIR with TBI-based regimen in MRD positive status patients was observed. These findings could drive changes in local practice guidelines where TBI is not feasible for all ALL patients, suggesting that TBI may be preferentially reserved for patients with positive MRD prior allo-HSCT in this setting. Blinatumomab has demonstrated promising results in clinical trials, showing improved rates of MRD clearance and increased overall survival in patients with MRD-positive B-ALL prior to allo-SCT.^10^

The 5-year OS and EFS were also not significantly different in the F-TBI and CT groups. Most previous studies reported better survival with TBI-based.^2^ Eder et al demonstrated that EFS was significantly better with TBI in patients transplanted in CR1 compared to those transplanted in subsequent CR.^3^ We expected to have survival improvement in TBI group given the significant reduction of relapse risk in this group and similar NRM in both groups. The absence of significant differences in survival might be explained by a higher number of patients with Ph+ ALL in CT group. All patients who had molecular relapse were treated with tyrosine kinase inhibitors (TKIs) and the majority of these patients are alive.

The prognosis of Ph+ ALL has significantly improved with the introduction of different-generation of TKIs and the standard indication of allo-SCT in Ph+ adult ALL patients is being reconsidered for patients in CR1, particularly who achieved negative MRD status after induction/consolidation therapy with chemotherapy-free treatment like ponatinib and blinatumomab combination.^11^ Also, the high-risk of Ph+ ALL has been reduced, particularly when using post-transplant TKI maintenance as prophylactic regimen in all patients regardless of MRD status once hematopoietic recovery is achieved or as preemptive MRD-triggered intervention.^12^

Our study has some limitations. It is retrospective which leads to missing data (eg, pre-allo MRD), including a small number of patients. The study was heterogeneous in terms of leukemia phenotype, including both B-cell ALL and T-cell ALL, with patients with Ph+ ALL. Furthermore, the population was diverse in MRD status. The CT group itself was also heterogeneous, comprising various treatment regimens and protocols. However, most of patients were transplanted in CR1 and all TBI patients received the same scheme total dose of F-TBI and the same dose rate.

In conclusion, our retrospective study showed that once-a-day F-TBI-based conditioning regimen in HLA-MSD transplant for adult ALL was associated with significantly lower risk of relapse than CT-based regimen with comparable NRM and better OS and EFS. There was a trend to better outcomes with TBF within CT group and benefit TBI in MRD positive status patients in terms of CIR and survival. Furthers prospective studies comparing TBI-based regimen to homogeneous group of CT are needed to validate this finding.

## CONFLICTS OF INTEREST

The authors declare they have no conflicts of interest.

## AUTHORS’ CONTRIBUTION

Conceptualization: Nour Ben Abdeljelil (Equal), Rihab Ouerghi (Equal). Methodology: Nour Ben Abdeljelil (Equal), Saloua Ladeb (Equal), Tarek Ben Othman (Equal). Writing – original draft: Nour Ben Abdeljelil (Equal), Rihab Ouerghi (Equal). Writing – review & editing: Nour Ben Abdeljelil (Lead). Formal Analysis: Rihab Ouerghi (Equal), Insaf Ben Yaiche (Equal), Rimel Kanoun (Equal). Investigation: Rihab Ouerghi (Equal), Insaf Ben Yaiche (Equal), Rimel Kanoun (Equal). Data curation: Sabrine Mekni (Equal), Dorra Belloumi (Equal), Ines Turki (Equal). Visualization: Lamia Torjemane (Equal), Chiraz Nasr-Ammar (Equal). Supervision: Saloua Ladeb (Equal), Tarek Ben Othman (Equal). Validation: Tarek Ben Othman (Lead).
